# Life after cancer treatment – existential experiences of longing

**DOI:** 10.1080/17482631.2020.1838041

**Published:** 2020-10-28

**Authors:** Venke Ueland, Kristine Rørtveit, Elin Dysvik, Bodil Furnes

**Affiliations:** a Faculty of Health Sciences, University of Stavanger, Stavanger, Norway; b Researcher of Psychiatric Nursing at Department of Research, Stavanger University Hospital, Stavanger, Norway

**Keywords:** Cancer survivor, existential experience, phenomenological hermeneutics, longing, uncertainty, lived experience, lifeworld

## Abstract

**Purpose:** The study aimed to gain insight into existential longing as experienced by people treated for cancer.

**Method:** An exploratory phenomenological–hermeneutical design was used, and data were collected through in-depth interviews with 21 people recruited from a cancer organization.

**Results:** Three themes emerged: longing to be oneself, longing for relief from suffering, and longing for rootedness. The theoretical understanding of well-being developed by Todres and Galvin was used to illuminate how the life-fulfilling power of longing is inherent in dwelling–mobility.

**Conclusions:** During the theoretical interpretation and discussion of these findings, a new analytic step revealed a state of uncertainty that can influence longing. The findings of this study may help fill the gap in the current health-care approach to cancer survivors by highlighting the importance of a new professional perspective of listening to patients describe their existential burden. Such an approach may create greater clarity and thereby allow longing to flow more freely towards future possibilities and well-being.

## Introduction

There seems to be limited research about longing as experienced after cancer treatment (Ueland et al., 2018). Longing might provide a fruitful perspective (Ahrberg et al., 2010) for understanding an individual’s unique existential cancer journey and for improving holistic care (Ellingson & Borofka, 2020; Hvidt, 2017; Mikkelsen et al., 2008). Thinkers within philosophy, religion, and psychology have written about how elusive the phenomenon longing seems to be because it is deeply rooted in the existential world of humans (Dalton, 2009). Longing can also be a personal experience that belongs to human existence within health and suffering. Listening to a person describe longing might be helpful for unifying a complete life by addressing both loss and the possibility of moving towards the processes of reconciliation (Ueland et al., 2015).

Cancer survivorship has been conceptualized as the period after a traumatic event that alters a person’s perceptions of self, the world, and possible futures (Peck, 2008; Tindle et al., 2019). Long-term survival after cancer has increased with advances in early diagnosis and treatments. About half of patients diagnosed with cancer will survive for 10 years or more (Lagergren et al., 2019). There is a need for increased knowledge about the lived existential experiences related to the disease and its treatment (Hvidt, 2017; Knox, 2018; Mikkelsen et al., 2008).

Some studies have claimed that going through cancer treatment requires the person to face a new reality that is shaped by the side effects and late effects of treatments such as damage caused by chemotherapy, radiation, surgery, medications, and other treatments (Ellingson & Borofka, 2020; Knox, 2018; Seiler & Jenewein, 2019). The resultant suffering may be reflected as physical, cognitive, emotional, sexual, or social struggles. In addition, after cancer treatment, the person may experience restricted capacity in everyday life caused by emotional distress, depression, anxiety, sleep problems, and fatigue (Ellingson, 2017; Ellingson & Borofka, 2020; Jakobsen et al., 2018; Knox, 2018; Seiler & Jenewein, 2019).

Studies have shown that, in addition to various side effects and late effects, cancer can induce irrevocable experiences of oneself as different according to personal identity, which may lead the patient to strive to find a firm footing in their sense of self (Knox, 2018; Little, Sayers et al., 2000; Tindle et al., 2019). The journey after cancer treatment is a critical time for many because the biomedical treatment may leave the patient feeling alienated within his or her lived body (Hvidt, 2017). To be alienated in this way is to experience the world as no longer the same and in which the person exists in a state of liminality in between different living spaces (Little, Paul et al., 2000).

Much of the research on life after cancer treatment is limited to symptom-based measures focused primarily on psychosocial needs, particularly anxiety and depression (Tindle et al., 2019). To our knowledge, few research studies have focused on the embodied existential lived experiences associated with life after cancer treatment (Ellingson, 2017; Ellingson & Borofka, 2020; Hvidt, 2017; Knox, 2018, 2020; Mikkelsen et al., 2008; Tindle et al., 2019). Our study was initiated to obtain knowledge about existential longing and to initiate a discourse for people after cancer treatment that is more broadly representative of this group (Tindle et al., 2019). A life phenomenon such as longing is important to the existential life and is often overlooked by health-care professionals (Delmar, 2006). Landstad and Åhrberg claim that longing encompasses ideas, emotions, and values that influence people’s self-awareness and view of their situation (Landstad & Åhrberg, 2018).

We suggest that it may be fruitful to focus on longing, which is a personally authentic phenomenon. Following the research mentioned above, in this study we explored the experiences of people after cancer treatment from their perspective to capture the phenomenon of longing. We suggest that a lifeworld perspective may be beneficial by focusing on longing.

This approach requires consideration of the term cancer survivor because research has demonstrated that it does not capture the individual’s lived experiences (Berry et al., 2019; Ellingson, 2017). In this paper, we use the words often mentioned by the participants in this study because these terms better reflect the lived experience of survivors, such as *treated for cancer, beyond cancer*, and *life after cancer treatment*.

### Aim

This study aimed to obtain insight into existential longing as experienced by people treated for cancer.

### Theoretical perspective

People living after cancer treatment face physical, social, and existential burdens and, like others who have experienced trauma, they seem to be challenged by the experience of well-being. Eriksson defines health as soundness, freshness, and well-being (Eriksson, 2018). Longing may be powerful because it involves the search for something significant that helps one achieve health and well-being. A person can experience moments of well-being even while striving to live one’s life (Ueland et al., 2015). From a caring science perspective, well-being is a subjective experience that is closely related to longing, which represents an expression of a person’s unique inner world (Eriksson, 1994; Ueland et al., 2018).

Todres and Galvin’s existential theory of well-being as dwelling–mobility is characterized by the unity of two experienced modes—“dwelling” as the feeling of being at home in the present and “mobility” as the feeling of unrest when searching for future possibilities (Todres & Galvin, 2010). Todres (2004, p. 2) claims that the “wound of longing” might be a gate to some of our deepest existential possibilities because longing touches our human concerns to a degree that has been called “spiritual” or “ontological.” Todres (2004) provides a strong connection between longing and moving towards well-being. The dwelling–mobility theory can be used to extend the understanding of a patient’s lived experiences of longing after cancer treatment.

When experiencing health challenges, uncertainty is a dynamic experience that may have a major effect on the patient’s experience related to illness (Mishel, 1990). This understanding is consistent with the findings of research into cancer patients’ experiences throughout their cancer journey. The picture of patients’ diverse and dynamic experiences of uncertainty in the different stages of their illness trajectory is complex (Dauphin et al., 2020; Hall et al., 2014). Uncertainty may affect a patient’s sense of control, direction in life, and structure of equilibrium (Hansen et al., 2012).

## Methodology

Our study used an exploratory phenomenological–hermeneutical approach involving qualitative interviews (Gadamer, 2007; Galvin & Todres, 2013). A lifeworld approach is advisable for gaining a deeper understanding of the phenomenon of longing by investigating the first-person perspective on the phenomenon (Dahlberg et al., 2008) and considering the person’s experiential “views from inside” (Galvin & Todres, 2013). According to Gadamer (2007), meaning is contextual because people understand themselves through the worlds in which they live. In our study, hermeneutical understanding means that longing is interpreted in the interplay of both the whole and parts in the hermeneutic circle.

### Data collection

We recruited participants among adults who had experienced cancer treatment and had been in contact with a local Cancer Association in the south of Norway in 2019. A purposive sample was suitable because the participants are proficient and well informed of the phenomenon of interest. We received an overwhelming response from people who were willing to talk in detail and decided to take anyone who agreed. The age range of these participants was 28–72 years, and there was a reasonable balance of sex (6 men and 15 women) (Table I). The participants had all been affected by different types of cancer and treatments: nine had breast cancer, four had prostate cancer, two had colorectal cancer, two had gynaecological cancer, two had testicular cancer, one had Hodgkin’s lymphoma, and one had non-Hodgkin/CNS lymphoma. Most had finished cancer treatment 4 years before the study.

**Table I.** Background Information of the Participants who have received cancer treatment

Sex	Age (Years)	Occupation	Year of Treatment Completion
Female	35	Working	2016
Female	38	Sick leave	2019
Female	40	Working	2015
Female	42	Working	2018
Female	48	Working	2015
Female	50	Disability benefit	2017
Female	50	Working	2018
Female	52	Working	2015
Female	56	Sick leave	2016
Female	57	Working	2017
Female	57	Working	2009
Female	60	Working	2017
Female	65	Disability benefit	2016
Female	72	Retired	2015
Female	72	Retired	2017
Male	28	Working	2016
Male	42	Working	2010
Male	53	Working	2012
Male	53	Working	2016
Male	58	Disability benefit	2018
Male	64	Retired	2018

There is no correspondence between the table listing and the number in the findings. (N = 21).

The inclusion criteria were that the participant had undergone cancer treatment, was feeling healthy at the time of the study, was motivated to participate as an individual in an in-depth interview, and was willing to share their experiences. One patient had recently been informed about cancer metastases but was still interviewed because of his experiences of life after cancer treatment.

Participants were invited to join the study through the Cancer Association leader who provided written information to those who were in contact with the Cancer Association. Interested people contacted the first author directly to ensure anonymity. After agreeing to join the study, each participant contacted the first author to plan the location and time for the interview.

The interviews were led by the first author in a meeting room at the Cancer Association’s offices and lasted for 60–90 minutes each. An interview guide was used with an open approach in the in-depth interviews, and nuances were explored by asking follow-up questions. The interviews always started with the same line, “Please tell me about your experiences of longing as experienced by you after being treated for cancer.” The follow-up questions focused on the participant’s thoughts and feelings related to longing in the context of their post-cancer experiences. The term longing was not explicitly defined for the participants, and the interviewer was open to the individual participant’s notion of what longing means without giving a specific definition. The interviews focused on the participants’ existential situation. Openness towards the subject, descriptions of the topic, and an attempt to disregard prior knowledge were central to the approach. The interviews were audiotaped and transcribed verbatim, and the transcriptions provided the empirical data material for this study.

### Data analysis

The text was analysed and interpreted on the self-understanding level, commonsense level, and finally theoretical level of understanding (Table II) (Kvale & Brinkmann, 2009). This process was consistent with the hermeneutical movement between the particular and the whole. The researchers’ preunderstanding was derived from a theoretical and clinical nursing perspective, which was based on the belief that longing is an essential existential phenomenon for a person struggling with health issues. All authors were involved in the interpretation and all levels of analysis. The entire text was read and reread, an intuitive initial holistic understanding was derived, and fragments of significance emerged.

**Table II.** Examples of three contexts of interpretations

Three levels of interpretations		
Self-understanding	Common sense	Theoretical interpretation
I would have longed to be normal, with no limitations to my life … My longing would be that it never happened … I just want to be myself. … There’s a grief of losing myself in a way … I don’t feel quite the same. There’s a new distance there.	*Longing to be myself*	Longing is intertwined in dwelling–mobility, a movement towards wellbeing. Uncertainty can be glimpsed which have impact on longing. Living in a condition of uncertainty might suppress the movement of longing towards future possibility. Reducing the uncertainty create more clarity and thereby might relief longing to flow more freely towards well-being.
My longings? I hope my pains gradually will let go … … When I wake up it’s so painful that I can just forget about going back to sleep again. One day without pain. That would’ve been fantastic! I don’t need a month. I need one day!	*Longing for relief from suffering*	
My longing is to be healthy … You live with a small shadow next to you … The fear of getting cancer again is much more present now, since I’ve had cancer and have seen that my body is capable of producing it (cancer) … I actually have great problems thinking ahead. It’s uninteresting in a way.	*Longing for a safe place in time.*	

#### Self-understanding

The co-authors participated in further reading, analysis, and discussion to reach consensus. First, the interpretation aimed to reveal the participants’ self-understanding. Next, meaning units were organized and coded into small headings and arranged under preliminary headings.

#### Common sense

The participants’ self-understanding was reformulated, a new level of abstraction emerged, and the units in the text were systematized. This was visualized as interconnected topics. In this process, new levels of abstraction took form and yielded the three themes that are presented in the Results section.

#### Theoretical interpretation

This interpretation is based on relevant theories and previous studies of the subject. The relevant theories that may deepen understanding of the findings are presented in the Theoretical interpretation and discussion section (Kvale & Brinkmann, 2009).

### Ethical considerations

The study was approved by the Ethical Committee and by the Norwegian Social Science Data Services (no 2018/2278). The participants provided their consent after receiving written information about the project. Confidentiality was guaranteed, and information mentioned the right of participants to withdraw from the study without needing to give any reason. Deidentified data have been stored in line with strict procedures at the University of Stavanger and the Ethical Committee and access is limited to the first author. The participants were informed that they had access to support from professionals at the clinic, if needed, after the interviews.

## Findings

The following findings represent the participants’ descriptions of their existential experiences of longing after cancer treatment. The participants were invited to reveal their longings by acknowledging their life as it feels at present. Based on the *self-understanding level* and *commonsense level*, the following themes were analysed: Longing to be oneself, Longing for relief from suffering, and Longing for rootedness.

We present the findings in the form of quotes by the participants as a way to give voice to their self-understanding. Following each theme, we offer an interpretation at the commonsense level. Longing is evident in the empirical data and the participants’ statements.

### Longing to be oneself

All participants experienced that life has changed and that they are living a different life. This experience creates a longing for oneself as the participant once was.

(Participant 3) I’m not really sure if I’m longing for much … I feel I’ve found the formula … that I’ve reached a calm that makes me have a good life. I long only for being left in peace and being with myself … And, of course, it depends on what kind of values you have in life and what you want to spend your resources on. (Participant 8) I’ve come to know myself better. … learned about my inner resources. (Participant 9) Yes, there’s a new inner life; it was there all the time, maybe, but it has become more apparent.

The participants expressed a longing for self but at the same time satisfaction with themselves. They have reached a deeper understanding of themselves, and their longing rests within a clarified frame of life. Although more restricted than before, life seems to be good.

Some participants commented that their social life has become more confined and that they found it challenging to accept they are living a more withdrawn existence.

(Participant 14) What I reacted to when it was over, and I’d become cancer free, were people’s expectations. They were probably thinking, “Now that you’ve recovered you can move on with your life.” However, you can’t just move on. (Participant 3) Meeting up with people is something I don’t need at all … I will not spend my days on such things … What I yearn for is not having to justify this to myself. … And maybe being more open about it, so that people find the courage to comment on it. (Participant 21) Other people look upon it as whining, that’s why it’s comforting to talk about it to someone who knows what it feels like to be tired and worried, and what it costs to go through this illness. And who can say, “Yippee for being alive”—that’s part of it, too. However, there’s always a “but” afterward.

The participants noted a longing for others to recognize the changes in their lives. They ask themselves whether the people they interact with really acknowledge the changes. Although they feel good about the limitations on their social lives, at the same time, they feel the outside world is not yet capable of meeting them where they are. Discerning between inner and outer voices may be difficult.

Being afflicted with cancer, and going through treatment, has left some scars. Life will never be the same, and participants struggle persistently to find themselves and to reach self-understanding.

(Participant 8) I long for a life that resembles a more normal life, like it was before. (Participant 2) You don’t return to the person you were before. Too much has happened to make this possible. (Participant 13) I would have longed to be normal, with no limitations to my life. To be able to do things, to feel passionately about something. Because when I really go in for something, … it’s a strain on my body, my body doesn’t tolerate it, committing to things the way I did … (Participant 14) Perhaps tiredness is going to stay with me for the rest of my life, so that I may never get back to what I was. You know you’ve become a limited person. (Participant 8) One thing I long for would be to go hiking in the mountains again. That’s a different kind of soreness. I guess that after a crisis, you will either get better or bitter, at least that’s what some people say. In some ways, I’ve become better. But at the same time, there’s so much grief to cope with. I think you cannot embrace all the grief at once. You need to attack bits and pieces. (Participant 17) My longing would be that it never happened, but of course, that’s only wishful thinking. I just want to be myself. … There’s a grief of losing myself in a way. It is as if I’ve lost some of my self-confidence. I don’t know. … My view of myself as well. … I don’t feel quite the same. There’s a new distance there.

The participants expressed longing for oneself; that is, after cancer, one looks on and experiences oneself as different. The longing relates to being ordinary or normal and living everyday life as before. Illness has imposed restrictions on their way of life, and it is impossible to revert to where they once were. Living with tiredness limits living one’s life fully. Fatigue or tiredness can prevent one from engaging in life so that one might feel limited as a human being.

### Longing for relief from suffering

The question, “What are your longings?” also evoked the pain and suffering experienced by some participants. Common to these extremely taxing experiences is that suffering restricts the life being lived now. The participants expressed a longing for relief from suffering and to be able to live their life as desired.

(Participant 9) My longings? I hope my pains gradually will vanish … so that I can rest my head on the pillow and sleep for one whole night. Being able to rest and wake up fully rested. (Participant 16) Where shall I start? I have prickling and tingling sensations in my arm … as a result of that, sleep has been reduced to a maximum of 2.5 hours a day. I wake in pain, … When I wake up, it’s so painful that I can just forget about going back to sleep again. One day without pain. That would be fantastic! I don’t need a month; I need just one day! Number two on my list is being able to play with MM (child) the way I wish to, it … sorry (tears). This is a very strong hope and wish for me … Being fully able to be the parent I want to be. (Participant 17) I guess the longing is for me to be myself … The pills make me feel I’m shrouded in a blanket. I do a lot of fun things, but that doesn’t always make me happy. What really makes me happy is when I’m happy in myself. And sometimes, if the dark blanket covers me, … the potential for being happy in myself just disappears. Because then everything becomes so complicated and difficult.

These participants expressed a longing for relief from their pain and to have a proper rest. The damage to their body caused by their illness restricts their potential to live a life in a manageable way without suffering. They long for their pain and discomfort to be alleviated so they can live life fully.

Several participants sensed their losses acutely in relation to relationships, sexuality, and self-image. Their experience of self has changed because of weight gain, menopause, impaired potency, and an altered sexual self-image. Both women and men said they feel less attractive. Some women point to challenges in the way they look upon themselves.

(Participant 21) Am I attractive? Am I good looking? I’m going to convince myself, and then him, that I’m still attractive. That I’m not damaged goods. (Participant 1) I’ve put on a lot of weight as well. It’s not cool at all. (Participant 18) My body feels stiff and old. You feel you’re not at all attractive. (Participant 17) After my illness, I’ve become much more self-centered. I don’t like that. To be honest, I feel that the treatment has made me ugly; my self-image—my looks, my body, the feminine me.

Changes in sexuality and body image influenced the way the participants feel about and look upon themselves.

(Participant 5) I guess my longings relate to having something to contribute … in a relationship and in married life. I’m struggling to adapt to that. I’ve had ups and downs where I’ve wanted to walk out on the relationship … I feel that I’m living in a shared house rather than being in a relationship. (Participant 17) I’ve put my life on hold. That goes for my sexual life, too … I just long to be normal and for things to work out … I guess that is the worst part.

The participants commented on their longing to be able to live as a woman or man without suffering and loss of self. The losses have caused them to put their life on hold. They expressed sorrow relating to the changes in sexuality and their self-image as a woman or man. Their changed body affects their intimacy with their partners or those they live closely with. They long to alleviate the burden of not being able to experience sexuality as before.

### Longing for rootedness

All participants mentioned the future, present, and past when invited to talk about their longings. It is a challenging longing for rootedness in the tension between the past as it was and an uncertain future. Their fundamental longing is to be healthy.

(Participant 2) I know perfectly well I may become ill again. Being afflicted with cancer is a threat to a continued lease on life. (Participant 18) My longing is to be healthy. I think this is the fundamental longing. You live with a small shadow next to you. You’re living with it, but you don’t let it take too much space … If I’m very tired, it will hit me harder, or if I feel very well, it suddenly becomes much more threatening. (Participant 9) I hope I’ll not experience a relapse. The fear of getting cancer again is much more present now since I’ve had cancer and have seen that my body is capable of producing it (cancer).

Their longings relate to remaining healthy in the future. Having suffered from cancer has meant that the finality of life has become closer. Although they no longer have cancer, they are constantly reminded it may return. Participants live in a constant state of uncertainty in which they recognize themselves as different.

Several participants said that having cancer makes it difficult to plan ahead.

(Participant 2) I actually have great problems thinking ahead. It’s uninteresting in a way. It’s the here and now that counts. (Participant 13) I don’t really long for anything at all. I have only hope and faith. … You may easily get lost when you long for things. It becomes so intangible. Longing is something one has been—something one wishes to return to. Or you can long for things in the future. My longings look backward, maybe. Faith and hope—that’s what you need, but longings, I don’t know about that. To me, it’s not the most positive word, but sore—to long for something. (Participant 15) That’s the problem. I don’t long for anything, apart from wishing that I could long for something. Like waking up in the night craving an ice cream. Or wanting to go to the cinema. Or going on a vacation. But I’ve put a lid on all that to be on the safe side. To avoid disappointment, you know.

The participants experience their longing for the future differently, and several are struggling with their tentative relationship with this longing. Some noted that they may not focus on longing for a future as much as in the past. The participants have put their longings on hold, although they may long for the future at another time. It is challenging to deal with their longing because they do not know whether the future they long for is possible. Some participants harbour longings that seem endless and impossible.

(Participant 21) After some time, after chemotherapy or the cancer, … I start to fear that the illness will come back. … I get so impatient with the rest of my life … I don’t need a vacation; I just want my everyday life to be somewhere else … Sometimes I get desperate inside. I become very sad because I feel I’m not satisfied with my current life even though everything’s okay. But, I’m in a hurry, and that’s probably mostly because I don’t have the same time perspective as everybody else; I dare not hope for that … This has created additional unrest. I don’t want to die without having done more in my life. I must fulfill my longing … (Participant 17) My longing would be that it never happened, but that’s wishful thinking … (Participant 4) I long for the life I had before, when I was surrounded by everybody I love, when I could go home to my mum and dad.

When the participants opened up to talk about their longings, they revealed longings that have no boundaries. They longed to be somewhere else, away from the here and now, in another time, another place. They felt that they might become more rooted in life in another place. They commented that this longing is like looking for oneself, without finding oneself where one actually is. The participants longed to be able to catch up with life more. Their ultimate longing was directed towards their dreams and appeared as a boundless longing. There seemed to be a longing for a past that they no longer have access to.

## Theoretical interpretation and discussion

This study aimed to gain insight into longing as experienced by people having been treated for cancer. Our analysis of their perspectives showed three main themes: *Longing to be oneself, Longing for relief from suffering*, and *Longing for rootedness*. Through a new analytic step, the themes revealed a state of uncertainty that affects their longing. Uncertainty was apparent as they were looking for a new foothold in life. Because they had suffered from various side effects and late effects and questioned whether life will be the same as before, they did not dare to long for a more livable future. Such uncertainty seems to relate to all the changes they had experienced in life as a result of having cancer.

When seen through the lens of longing, the perspectives of these cancer survivors reveal that burdensome existential experiences remain after cancer treatment. The existential challenges seem to relate to changes in self-knowledge, self-perception, everyday life, relationships, thoughts about the future, and different health challenges. This state of uncertainty seems to involve questioning the basic perceptions of oneself. This is consistent with the description by Hansen et al. of the uncertainty related to being on constant alert and the feeling that life may be unpredictable because it is influenced by health and an unknown future (Hansen et al., 2012).

Todres and Galvin’s elaboration of longing as a significant power in dwelling–mobility sheds some light on the understanding of how uncertainty affects longing. Dwelling–mobility is part of a dynamic movement and is experienced as an openness to a simultaneous longing for stability and peace while also longing for adventure and expanse (Galvin & Todres, 2013; Todres & Galvin, 2010). This reflects that longing is intertwined with dwelling–mobility.

By looking first at *dwelling* as one side of dwelling–mobility (Todres & Galvin, 2010), we understand that dwelling means recognizing one’s situation, acknowledging what is there, and then finding oneself within that situation, which becomes a sense of rootedness. Everyday life might be a form of “being at home,” which might provide a protective refuge. Dwelling can also refer to attunement to one’s existence and “coming home” to one’s situation (Galvin & Todres, 2013).

It is this sense of rootedness, a feeling of “being at home” and finding refuge in everyday life, that seems to be a challenge after cancer treatment. Everyday life with the context of a dwelling place may no longer seem to be rooted. This existential change has also been reported by others (Hvidt, 2017; Knox, 2018; Little, Sayers et al., 2000; Tindle et al., 2019). Existential experiences after cancer treatment can be understood as a disruption of the lifeworld (Ueland et al., 2020). The participants in our study perceived a kind of loss of rootedness, which appeared as their uncertainty related to recognizing themselves as different compared with before their cancer treatment. The uncertainty noted by these participants reflected their realization that their life has changed but it may be unclear how and what their current life has become. This observation was noted by Dauphin et al. (2020), who claimed that when primary treatment ends, patients enter a transition period in which they must adapt to a new life. This was also reflected in our study in the challenge for patients learning how to live with the significant burden of the cancer journey and with the possibility of recurrence.

It seems that uncertainty can blur and complicate the feelings of being at home in oneself and in the present life because of the loss of the sense of refuge in one’s present everyday life (dwelling). This seems to narrow the living space because the uncertainty in everyday life can suppress the dynamic power of longing. Because one does not feel at home in one’s life, there might not be any firm footing from which to start one’s longing. It may also be difficult to see clearly what life is like and what one longs for because life feels different and there are significant health challenges to handle in everyday life. As observed in other studies, living with uncertainty may lead to a feeling of otherness or something unknown, which cancer survivors may feel unable to avoid or manage (Ellingson & Borofka, 2020; Hvidt, 2017; Knox, 2020). At its extremes, uncertainty may increase the burdens of life, and being uncertain about oneself and one’s place in the world may threaten one’s existence (Peters et al., 2017; Van Den Bos, 2009).

The other movement towards well-being—*mobility* (Todres & Galvin, 2010)—can be described as the power to let go of something as it was before and search for the future. Mobility is a basic condition of life that carries a sense of unfinishedness that requires one to seek future possibilities (Todres & Galvin, 2010). Being able to let the vulnerable longing unfold can help to make life flow more freely towards well-being. Longing can provide the strength to move on and to expand one’s living space. This means that the movement of longing flows freely only when one can both find a place in the present (dwelling) and, at the same time, allow the power of longing to lead to the search for something that has not yet arrived (mobility) (Todres & Galvin, 2010). Then, longing might release the power of life and become a force for well-being (Ueland et al., 2018).

Our findings suggest that future mobility is, in some ways, restricted. There is a longing for rootedness, and the future is experienced as challenging and different. The participants do not seem to relate to the future in the same way as before having cancer. Longing for relief from suffering because of the major health challenges seems to overshadow life after cancer treatment and to limit a person’s ability to look ahead. The participants in our study mentioned having a closed-off relationship with the future because of the losses, limitations, and worries about recurrence as part of the cancer journey. We also found that longing was restricted by not longing for anything in the future but instead longing for the life lived before having cancer—even as far back as childhood.

Longing for a former life before cancer seems to reveal a struggle with dwelling–mobility (Galvin & Todres, 2013; Todres & Galvin, 2010). Longing for the past makes one more vulnerable because this is a longing for what is unattainable (Ueland et al., 2018). When longing focuses on the past, one can become fixed in an isolated state of mind, and life seems to lack continuity and does not pulsate and flow freely (Todres, 2004). Our interpretation of our findings is that it may be difficult to relate to the future if one does not find refuge in one’s present condition. This view is consistent with Dauphin et al.’s view (2020) that experiencing uncertainty after cancer treatment shapes the individual’s perspective on the present and the future, and positions that person in an ambiguous relationship with time. When this happens, the past can seem too close yet the future is receding, which can lead to the feeling of being “frozen in time” (Galvin & Todres, 2013, p. 101), as if the future has been locked away. Cancer survivors seem to be anxious about how the future will be affected by cancer treatment because illness can be experienced as a continuous effort to find a new sense of equilibrium in life (Dauphin et al., 2020). If longing remains mainly retrospective, loss and melancholy are given more room than longing for future possibilities (Ueland et al., 2018).

Uncertainty seems to suppress longing. Acknowledging uncertainty may allow one to be open to allow for longing and thus release the processes of movement to flow again (Ueland et al., 2018). This could become possible by introducing space for self-reflection and self-knowledge. Longing connects people to time as they dwell in the past, present, and future (Ueland et al., 2018). Through one’s innermost longing, it might be possible to experience a transition and slow transformation of self as a long-lasting inner movement (Galvin & Todres, 2013). This suggests that people may be able to move beyond the experience of cancer through self-reflection and an awakening of selfhood (Knox, 2020). This movement could be facilitated by acknowledging one’s uncertainty as caused by the losses and embodied health challenges, and using this self-knowledge to move beyond the uncertainty and, hopefully, towards a more existential well-being. This flow is created by giving access to longing, encouraging self-reflection, and supporting the existential journey (Knox, 2018).

As noted by Dauphin et al. (2020), when treatment ends, patients enter a transition period in which they have to adapt to a new life. Cancer survivors talk about their situation as a period of enduring uncertainty, even after active treatment has ended. They seem to be living with disappointment about the fact their life and they had changed, which made it impossible to return to the way they were before cancer.

In our study, we saw glimpses of a breakthrough about uncertainty in some participants. Carefully using the processes of self-knowledge and self-awareness may help people after cancer treatment move towards an ongoing inclusion of what has been lost and changed, the challenges, and the potential to move forward.

Todres uses the poetic term “the wound of longing” when noting that humans are deeply vulnerable and touched by longing (Todres, 2004, p. 2). Nevertheless, honest and vulnerable longing provides a place where one can rest, and it is also about how one bears this longing and even finds it as a gate to some of one’s deepest existential possibilities (Todres, 2004, p. 2). Given that longing is intertwined in dwelling–mobility and as the movement towards well-being, living with uncertainty might suppress the movement of longing towards future possibilities. Listening to patients describe their longing may provide a path to finding refuge in the present as a foundation for expanding life towards the future. Reducing the uncertainty may create greater clarity, thereby allow longing to flow more freely towards well-being.

## Conclusion

The study results emphasize that after cancer, survivors are striving for a firm footing in the present and the future. Our study provides an existential perspective on well-being that may fill a gap in the current approach to the individual patient’s burden remaining after cancer treatment. The expressions of longing expressed by participants in our study reveal that living beyond cancer treatment is an uncertain condition. The theoretical understanding of well-being reveals how the life-fulfiling power of longing inherent in the dwelling–mobility movement is limited by the person’s condition of uncertainty. Our findings may help to reduce the gap in the current health-care approach to cancer survivors by highlighting how uncertainty can suppress longing and cause the patient to lose clarity and the potential to live fully in both the present and future.

Rehabilitation after cancer treatment should acknowledge the individual patient’s existential burden and challenges beyond cancer disease and treatment. Providing space for self-reflection and self-knowledge may promote the transformation processes towards greater well-being. Thus, expressing longing may help to promote relief from suffering, reduce uncertainty, and contribute to greater openness about the future.

When viewed from an existential perspective, the two important concepts within cancer survivorship, *longing* and *uncertainty*, may help to expand the understanding of ways to improve well-being for this group of people. Longing might reveal what really matters to the person after cancer treatment ends. Future research is needed to understand more about the existential experiences after cancer treatment through the lens of longing. Research is needed to understand fully the relationship between longing as an inherent power in dwelling–mobility and moving towards well-being, and how uncertainty can affect longing.

## Methodological considerations

One strength of our study is the interviews with 21 participants and the inclusion of both men and women of different ages who had experienced the individual burden of cancer disease and treatment. The information power appears to be strong, given that sample specificity provided adequate information and the data material comprised rich and detailed descriptions based on the study’s aim and established theory (Malterud et al., 2016). It is not possible to grasp the whole understanding of the phenomenon of longing because longing is implicit in the person’s inner world. It was therefore important that the phenomenon of longing not be defined in a limited way, although we found it important during the interviews to provide openings to elicit a rich description of the participants’ experiences of longing. The interpretation of longing in light of Galvin and Todres’ theoretical perspective on well-being has proven to be a suitable framework for synthesizing existing knowledge and for extending the sources of knowledge beyond the empirical interview data (Malterud et al., 2016).

A limitation of our study is that the biomedical background classified according to cancer type, treatment, late effects, and side effects or enduring damage do not relate to the findings. However, the purpose of the study was to describe the lived experiences of cancer survivors by fostering an openness towards the participants’ descriptions of their subjective experiences and not by using the more concrete biomedical approach. Another limitation of the study is that the approach to the interviews lacked a theoretical understanding or definition of longing. However, because this was an explorative study, we did not aim for complete descriptions of all aspects of longing, and we believe we have contributed substantially to in-depth knowledge about longing as experienced by people facing a challenging health problem (Malterud et al., 2016). The strong theoretical foundation relating to longing appeared after the data were analysed, as occurs when using the phenomenological approach. Another weakness is that there were more women than men in our study, and the women were slightly older than the men. Additional insights might be gained if there had been more men in the study.

We suggest that the insights into the phenomenon of longing shown here provide insight into the existential experiences after cancer treatment and allow for some degree of transferability by considering the culture and the context.

## Implications for practice

A new professional perspective is needed for health-care providers who offer individual post-cancer treatment. The processes of longing for life fulfilment rely on acknowledging and understanding oneself in a new situation. Our study reveals existential challenges after cancer treatment that require a better follow-up of this patient group. The establishment of rehabilitation services in which health-care professionals can take the time to follow the individual patient’s existential movement towards well-being beyond their cancer treatment would make a difference to patients. Hearing about the individual’s longings may help each patient create a personally authentic experience and to find a direction in life after cancer treatment. The emerging self-understanding of the individual’s unique existential cancer journey may help to clarify the issues and lessen uncertainty. Giving space for self-reflection and self-knowledge might provide the opportunity for the person to gain an authentic individual way to work towards future well-being. Health-care providers working in cancer rehabilitation should use a holistic approach by considering their patients’ existential burden. One-to-one counselling for the purpose of sharing the experience of longing is expedient. Confirmation of the state of uncertainty will help individual patients to leave behind what is impossible to reach, to live in the present, and to be open to the possibilities of the future.

## References

[cit0001] Ahrberg, Y., Landstad, B. J., Bergroth, A., & Ekholm, J. (2010). Desire, longing and vanity: Emotions behind successful return to work for women on long-term sick leave. *Work*, 37(2), 167–11. 10.3233/WOR-2010-1067 20938077

[cit0002] Berry, L. L., Davis, S. W., Flynn, A. G., Landercasper, J., & Deming, K. A. (2019). Is it time to reconsider the term “cancer survivor”? *Journal of Psychosocial Oncology*, 37(4), 1–14. 10.1080/07347332.2018.1522411 30614422

[cit0003] Dahlberg, K., Dahlberg, H., & Nyström, M. (2008). *Reflective lifeworld research* (2nd ed.). Studentlitteratur.

[cit0004] Dalton, D. (2009). *Longing for the other: Levinas and metaphysical desire*. Duquesne University Press.

[cit0005] Dauphin, S., Van Wolputte, S., Jansen, L., De Burghgraeve, T., Buntinx, F., & van den Akker, M. (2020). Using liminality and subjunctivity to better understand how patients with cancer experience uncertainty throughout their illness trajectory. *Qualitative Health Research*, 30(3), 356–365. 10.1177/1049732319880542 31617448

[cit0006] Delmar, C. (2006). The phenomenology of life phenomena–in a nursing context. *Nursing Philosophy: An International Journal for Healthcare Professionals*, 7(4), 235–246. 10.1111/j.1466-769X.2006.00282.x 16965305

[cit0007] Ellingson, L. L. (2017). Realistically ever after. *Management Communication Quarterly*, 31(2), 321–327. 10.1177/0893318917689894

[cit0008] Ellingson, L. L., & Borofka, K. G. E. (2020). Long-term cancer survivors’ everyday embodiment. *Health Communication*, 35(2), 180–191. 10.1080/10410236.2018.1550470 30466317

[cit0009] Eriksson, K. (1994). *Den lidande människan* [The suffering human]. Liber Utbildning.

[cit0010] Eriksson, K. (2018). *Vårdvetenskap: Vetenskapen om vårdandet - det tidlösa i tiden*. Liber Utbildning.

[cit0011] Gadamer, H.-G. (2007). *Sannhed og metode* [Truth and method]. Academica.

[cit0012] Galvin, K., & Todres, L. (2013). *Caring and well-being: A lifeworld approach* (1st ed.). Routledge. https://content.taylorfrancis.com/books/download?dac=C2011-0-08540-2&isbn=9781136181955&format=googlePreviewPdf

[cit0013] Hall, D. L., Mishel, M. H., & Germino, B. B. (2014). Living with cancer-related uncertainty: Associations with fatigue, insomnia, and affect in younger breast cancer survivors. *Supportive Care in Cancer*, 22(9), 2489–2495. 10.1007/s00520-014-2243-y 24728586 PMC6375671

[cit0014] Hansen, B. S., Rørtveit, K., Leiknes, I., Morken, I., Testad, I., Joa, I., & Severinsson, E. (2012). Patient experiences of uncertainty - a synthesis to guide nursing practice and research. *Journal of Nursing Management*, 20(2), 266–277. 10.1111/j.1365-2834.2011.01369.x 22380420

[cit0015] Hvidt, E. A. (2017). The existential cancer journey: Travelling through the intersubjective structure of homeworld/alienworld. *Health: An Interdisciplinary Journal for the Social Study of Health, Illness and Medicine*, 21(4), 375–391. 10.1177/1363459315617312 26589841

[cit0016] Jakobsen, K., Magnus, E., Lundgren, S., & Reidunsdatter, R. J. (2018). Everyday life in breast cancer survivors experiencing challenges: A qualitative study. *Scandinavian Journal of Occupational Therapy*, 25(4), 298–307. 10.1080/11038128.2017.1335777 28562163

[cit0017] Knox, J. B. L. (2018). Developing a novel approach to existential suffering in cancer survivorship through Socratic dialogue. *Psycho-Oncology*, 27(7), 1865–1867. 10.1002/pon.4750 29719084

[cit0018] Knox, J. B. L. (2020). Stories of despair: A Kierkegaardian read of suffering and selfhood in survivorship. *Medicine, Health Care and Philosophy*, 23(1), 61–72. 10.1007/s11019-019-09908-4 31144094

[cit0019] Kvale, S., & Brinkmann, S. (2009). *Det kvalitative forskningsintervju* [The qualitative research interview]. Gyldendal.

[cit0020] Lagergren, P., Schandl, A., Aaronson, N. K., Adami, H. O., de Lorenzo, F., Denis, L., … Ulrich, C. (2019). Cancer survivorship: An integral part of Europe’s research agenda. *Molecular Oncology*, 13(3), 624–635. 10.1002/1878-0261.12428 30552794 PMC6396379

[cit0021] Landstad, B. J., & Åhrberg, Y. (2018). Conceptualizing the driving forces for successful rehabilitation back to work. *Disability and Rehabilitation*, 40(15), 1781–1790. 10.1080/09638288.2017.1312569 28395536

[cit0022] Little, M., Paul, K., Jordens, C. F. C., & Sayers, E.-J. (2000). Vulnerability in the narratives of patients and their carers: Studies of colorectal cancer. *Health: An Interdisciplinary Journal for the Social Study of Health, Illness and Medicine*, 4(4), 495–510. 10.1177/136345930000400405

[cit0023] Little, M., Sayers, E. J., Paul, K., & Jordens, C. F. (2000). On surviving cancer. *Journal of the Royal Society of Medicine*, 93(10), 501–503. 10.1177/014107680009301001 11064684 PMC1298120

[cit0024] Malterud, K., Siersma, V. D., & Guassora, A. D. (2016). Sample size in qualitative interview studies. *Qualitative Health Research*, 26(13), 1753–1760. 10.1177/1049732315617444 26613970

[cit0025] Mikkelsen, T. H., Søndergaard, J., Jensen, A. B., & Olesen, F. (2008). Cancer rehabilitation: Psychosocial rehabilitation needs after discharge from hospital? A qualitative interview study. *Scandinavian Journal of Primary Health Care*, 26(4), 216–221. 10.1080/02813430802295610 18792854 PMC3406638

[cit0026] Mishel, M. H. (1990). Reconceptualization of the uncertainty in illness theory. *Image: The Journal of Nursing Scholarship*, 22(4), 256–262. 10.1111/j.1547-5069.1990.tb00225.x 2292449

[cit0027] Peck, S. (2008). Survivorship: A concept analysis. *Nursing Forum*, 43(2), 91–102. 10.1111/j.1744-6198.2008.00100.x 18447894

[cit0028] Peters, A., McEwen, B. S., & Friston, K. (2017). Uncertainty and stress: Why it causes diseases and how it is mastered by the brain. *Progress in Neurobiology*, 156(Sept), 164–188. 10.1016/j.pneurobio.2017.05.004 28576664

[cit0029] Seiler, A., & Jenewein, J. (2019). Resilience in cancer patients. *Frontiers in Psychiatry*, 10(Apr), 208. 10.3389/fpsyt.2019.00208 31024362 PMC6460045

[cit0030] Tindle, D., Windsor, C., & Yates, P. (2019). Centralizing temporality in adolescent and young adult cancer survivorship. *Qualitative Health Research*, 29(2), 173–183. 10.1177/1049732318797087 30182803

[cit0031] Todres, L. (2004). The wound that connects: A consideration of “narcissism” and the creation of soulful space. *Indo-Pacific Journal of Phenomenology*, 4(1), 1–12. 10.1080/20797222.2004.11433890

[cit0032] Todres, L., & Galvin, K. (2010). “Dwelling-mobility”: An existential theory of well-being. *International Journal of Qualitative Studies on Health and Well-Being*, 5(3), 5444. 10.3402/qhw.v5i3.5444 PMC2938924 20842215

[cit0033] Ueland, V., Dysvik, E., Rørtveit, K., & Furnes, B. (2020). Homeworld/Alienworld: A qualitative study about existential experiences after cancer treatment. *Scandinavian Journal of Caring Sciences*, scs.12902. 10.1111/scs.12902 32808312

[cit0034] Ueland, V., Nåden, D., & Lindstrom, U. (2015). Longing - A dynamic power in the becoming of health. *International Journal of Human Caring*, 19(4), 30–37. 10.20467/1091-5710.19.4.30

[cit0035] Ueland, V., Nåden, D., & Lindström, U. Å. (2018). Longing - a dynamic power in the becoming of health when suffering from cancer. *Scandinavian Journal of Caring Sciences*, 32(2), 924–932. 10.1111/scs.12527 28940617

[cit0036] Van Den Bos, K. (2009). Making Sense of Life: The Existential Self Trying to Deal with Personal Uncertainty. Psychological Inquiry, 20 (4), 197–217. 10.1080/10478400903333411 4 doi:10.1080/10478400903333411

